# An experimental approach to training interoceptive sensitivity: study protocol for a pilot randomized controlled trial

**DOI:** 10.1186/s12937-022-00827-4

**Published:** 2022-12-19

**Authors:** Petra Warschburger, Hanna R. Wortmann, Ulrike A. Gisch, Nadja-Raphaela Baer, Liane Schenk, Verena Anton, Manuela M. Bergmann

**Affiliations:** 1 NutriAct – Competence Cluster Nutrition Research, Potsdam, Berlin Germany; 2 Department of Psychology, Counseling Psychology, University of Potsdam, Karl-Liebknecht-Str. 24-25, 14476 Potsdam, Germany; 3 Charité – Universitätsmedizin Berlin, corporate member of Freie Universität Berlin, Humboldt-Universität zu Berlin, and Berlin Institute of Health, Institute of Medical Sociology and Rehabilitation Science, Berlin, Germany; 4 German Institute of Human Nutrition Potsdam-Rehbruecke (DIfE), Arthur-Scheunert-Allee 114-116, 14558 Nuthetal, Germany

**Keywords:** Digital intervention, Older adults, Interoception, Eating behavior, Intuitive eating, Partnership, Mindfulness, Randomized-controlled trial, NutriAct Family Study, Mixed methods

## Abstract

**Background:**

Eating in absence of hunger is quite common and often associated with an increased energy intake co-existent with a poorer food choice. Intuitive eating (IE), i.e., eating in accordance with internal hunger and satiety cues, may protect from overeating. IE, however, requires accurate perception and processing of one’s own bodily signals, also referred to as interoceptive sensitivity. Training interoceptive sensitivity might therefore be an effective method to promote IE and prevent overeating. As most studies on eating behavior are conducted in younger adults and close social relationships influence health-related behavior, this study focuses on middle-aged and older couples.

**Methods:**

The present pilot randomized intervention study aims at investigating the feasibility and effectiveness of a 21-day mindfulness-based training program designed to increase interoceptive sensitivity. A total of *N* = 60 couples participating in the NutriAct Family Study, aged 50–80 years, will be recruited. This randomized-controlled intervention study comprises three measurement points (pre-intervention, post-intervention, 4-week follow-up) and a 21-day training that consists of daily mindfulness-based guided audio exercises (e.g., body scan). A three-arm intervention study design is applied to compare two intervention groups (training together as a couple vs. training alone) with a control group (no training). Each measurement point includes the assessment of self-reported and objective indicators of interoceptive sensitivity (primary outcome), self-reported indicators of intuitive and maladaptive eating (secondary outcomes), and additional variables. A training evaluation applying focus group discussions will be conducted to assess participants’ overall acceptance of the training and its feasibility.

**Discussion:**

By investigating the feasibility and effectiveness of a mindfulness-based training program to increase interoceptive sensitivity, the present study will contribute to a deeper understanding of how to promote healthy eating in older age.

**Trial registration:**

German Clinical Trials Register (DRKS), no. DRKS00024903. Retrospectively registered on April 21, 2021.

**Supplementary Information:**

The online version contains supplementary material available at 10.1186/s12937-022-00827-4.

## Administrative information

Note: the numbers in curly brackets in this protocol refer to SPIRIT checklist item numbers. The order of the items has been modified to group similar items.

**Table:** 

Title {1}	An experimental approach to training interoceptive sensitivity: study protocol for a randomized controlled trial
Trial registration {2a and 2b}.	German Clinical Trials Register (DRKS), no. DRKS00024903.
Protocol version {3}	Protocol version 1.0 (01/26/2022).
Funding {4}	This research was funded by the Federal Ministry of Education and Research (FKZ: 01EA1806B). Open Access funding enabled and organized by Project DEAL (with funding by the Deutsche Forschungsgemeinschaft (DFG, German Research Foundation)- Project number 491466077.
Author details {5a}	Petra Warschburger^1,2^, Hanna R. Wortmann^1,2^, Ulrike A. Gisch^1,2^, Nadja-Raphaela Baer^1,3^, Liane Schenk^1,3^, Verena Anton^1,3^, Manuela M. Bergmann^1,4^ ^1^ NutriAct – Competence Cluster Nutrition Research, Berlin-Potsdam, Germany ^2^ University of Potsdam, Department of Psychology, Counseling Psychology, Karl-Liebknecht-Str. 24–25, 14,476 Potsdam, Germany ^3^ Charité – Universitätsmedizin Berlin, corporate member of Freie Universität Berlin, Humboldt-Universität zu Berlin, and Berlin Institute of Health, Institute of Medical Sociology and Rehabilitation Science, Berlin, Germany ^4^ German Institute of Human Nutrition Potsdam-Rehbruecke (DIfE), Arthur-Scheunert-Allee 114–116, 14,558 Nuthetal, Germany
Name and contact information for the trial sponsor {5b}	Federal Ministry of Education and Research (BMBF) 53,170 Bonn Germany Phone: + 49 (0)2289957–0 Email: information@bmbf.bund.de
Role of sponsor {5c}	The funding resource (BMBF / DFG (Open access publication)) has no role in the design of the study, the collection, analysis, and interpretation of data, and in writing the manuscript.

## Background

In recent years, research on intuitive eating has increased considerably [1]. Based on the approach of positive psychology [2], scholars have increasingly focused on identifying and promoting adaptive eating behaviors rather than taking the more pathology-focused approach of exploring disordered and maladaptive eating behaviors [3]. Intuitive eating is described as an adaptive eating behavior that is characterized by a strong physical connection with the body and eating in response to internal hunger and satiety cues, i.e., eating when hungry and stopping when satiated [4]. Previous research suggests substantial associations between intuitive eating and various physical and psychological health indicators [1, 5, 6], thereby supporting its adaptive properties [7]. Positive correlations were found between intuitive eating and life satisfaction, positive body image, self-esteem, positive affect [3, 4, 8], self-efficacy, and health-related quality of life [9]. Furthermore, intuitive eating is negatively associated with less healthy eating styles, such as restraint eating, emotional eating, and external eating, and eating disorder symptomatology [4, 9, 10] and positively related to healthy self-reported food intake [11]. Moreover, studies have shown a negative association between intuitive eating and body weight [7, 12, 13]. To date, research on the effects of intuitive eating on health outcomes has focused on younger and middle-aged adults [6]. Despite the scarce body of evidence concerning older age groups, first findings also indicate positive health-related outcomes in older age, such as lower restraint and lower BMI [14].

Primarily based on the perception of internal hunger and satiety cues [3, 4], intuitive eating is strongly connected to the concept of interoceptive sensitivity, which is defined as the ability to perceive and process internal bodily signals [15, 16]. Interoceptive sensitivity differs considerably among humans [17]. In addition, previous research has shown a positive relation between interoceptive sensitivity and intuitive eating [7, 18], especially with those facets of intuitive eating that are representing the reliance on hunger and satiety cues when eating and the willingness to eat for physical rather than for emotional or external reasons [7]. Moreover, interoceptive sensitivity was found to be lower in people with overweight and obesity [19–21] and to decline during aging [22]. It was also shown that the above-mentioned negative association between intuitive eating behavior and body weight status is mediated by interoceptive sensitivity, i.e., it can be explained by inter-individual differences in the ability to perceive and process internal bodily signals [7].

**Fig. 1:**
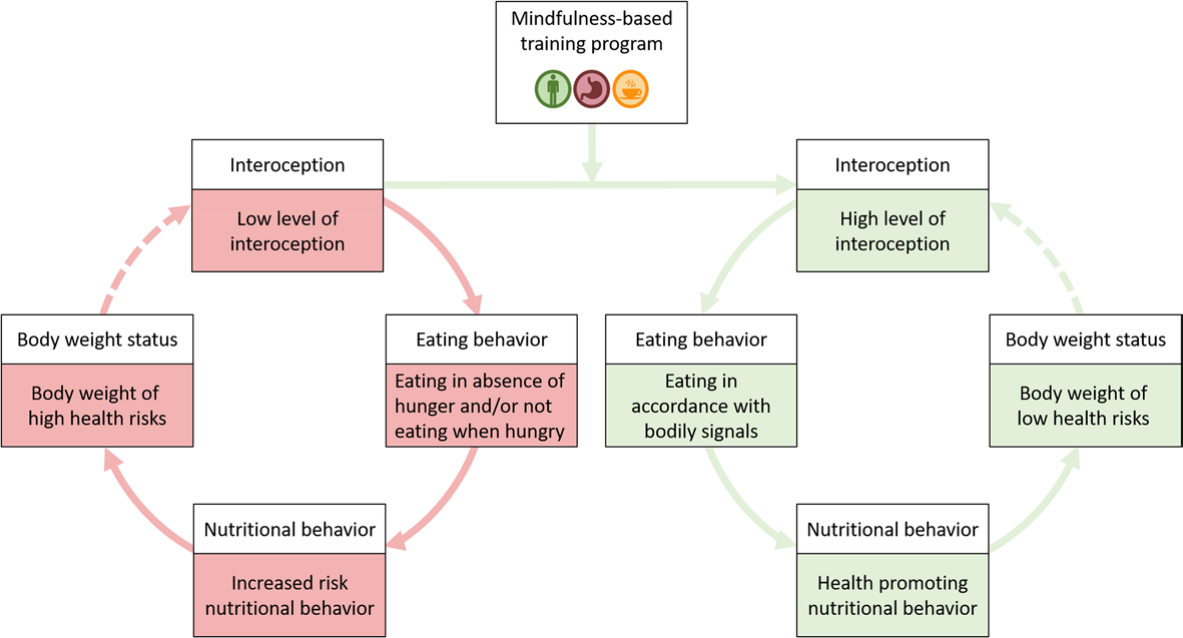
Theoretical Model

Figure 1 presents the theoretical model of the current study. Systematic reviews in recent years indicate that mindfulness-based interventions (MBIs) targeting eating behaviors can improve obesity-related eating behaviors, such as emotional eating, external eating, and binge eating [23–25] and support weight loss [24, 26, 27]. This underscores the role of mindfulness as a potentially beneficial component in treating obesity and promoting health-oriented eating [28]. Indeed, there is evidence that also the perception of internal bodily signals, such as the own heartbeat [29, 30] or feelings of hunger and satiety [31], can be improved through mindfulness-based training. Given the strong correlation between interoceptive sensitivity and intuitive eating, these findings suggest that training interoceptive sensitivity might be an effective method to increase eating in accordance with satiety and hunger signals and thus to promote intuitive eating. In the next step, intuitive eating might promote a positive nutritional behavior (e.g., a diet rich in plant-based foods such as whole grains, fruits, vegetables, nuts, and legumes), which in turn might lead to a body weight of low health risks. However, a recent systematic review and meta-analysis has shown that it remains unclear if a low interoception contributes to weight gain or in turn might be the consequence of weight gain [21]. Therefore, this path was represented with a dashed arrow in Fig. 1.

The increasing prevalence of overweight and obesity with age [32] and its associated health risks and comorbid conditions [33] make the age group of older adults an important target group for interventions designed to increase adaptive food choices and health-promoting eating. However, this age group is often neglected in research on eating behavior, with the majority of studies conducted on young adults [34]. Moreover, previous interventions targeting eating behaviors have predominantly focused on obesity-related outcomes and weight loss (e.g., [25, 26]). However, turning the focus to health-promoting eating itself as part of a health-oriented and mindful lifestyle appears to be a more holistic and therefore promising approach also regarding interventions aimed at promoting healthy aging.

Our health behavior should be considered in a social-ecological framework that stresses the complex interplay between individual and environmental factors [35]. Especially the influence of close social relationships must also be considered. It has been shown that individuals in close relationships influence the health-related behavior of their significant others [36]. In general, spouses’ health behaviors were found to be highly similar, with concordance among spouses increasing over the time of their relationship [37]. Moreover, it has been shown that a positive health behavior change in one partner tends to lead to a positive health behavior change in the other partner, i.e., persons are more likely to make a positive health behavior change if their partner does, too [38]. Indeed, intervention research indicates that targeting couples might enhance the effectiveness of health behavior change interventions compared to individual interventions [36, 39]. More specifically, emerging evidence also suggests benefits of targeting diet-related interventions at the couple level. So far, such intervention measures have proven to offer (cost-effective) alternatives to conventional individual-based strategies [38, 40, 41]. However, the body of literature remains sparse and most previous research activities have focused on diet-related conditions such as obesity or diabetes mellitus [36, 40]. Therefore, the need for more in-depth knowledge and further studies has been stressed [36].

### Aim of this study

Obviously, additional research is needed to gain a deeper understanding of how to promote adaptive eating in older age to enable healthy aging. The present randomized pilot study aims at investigating the following research questions and hypotheses: (1) Does the mindfulness-based training improve the perception of internal bodily signals in older age (primary outcome)? We hypothesize that the mindfulness-based training improves the ability to perceive internal bodily signals, i.e., participants of the two intervention groups show a more pronounced increase in their interoceptive sensitivity from pre-intervention to follow-up compared to participants of the control group. (2) Does training interoceptive sensitivity promote adaptive eating behavior and reduce maladaptive eating behaviors (secondary outcomes)? We hypothesize that the training promotes intuitive eating behavior and reduces maladaptive eating behavior (e.g., emotional eating, external eating, restrictive eating) from pre-intervention to follow-up, i.e., participants of the two intervention groups show a more pronounced increase in their intuitive eating and a more pronounced decrease in their maladaptive eating compared to participants of the control group. (3) Does targeting couples enhance the effectiveness of the mindfulness-based training (in terms of improving both interoceptive sensitivity and eating behavior) compared to targeting only one person in a partnership? We hypothesize that targeting couples enhances the effectiveness of the training, i.e., participants that train together with their spouses show a higher improvement of interoceptive sensitivity and eating behavior from pre-intervention to follow-up than those participants that are training alone. (4) How will the participants in the mindfulness-based training evaluate this approach? We will assess participants’ overall acceptance of the training, its feasibility as well as its medium-term impact and (non-) continuation in everyday life.

## Methods

### Study design

**Fig. 2:**
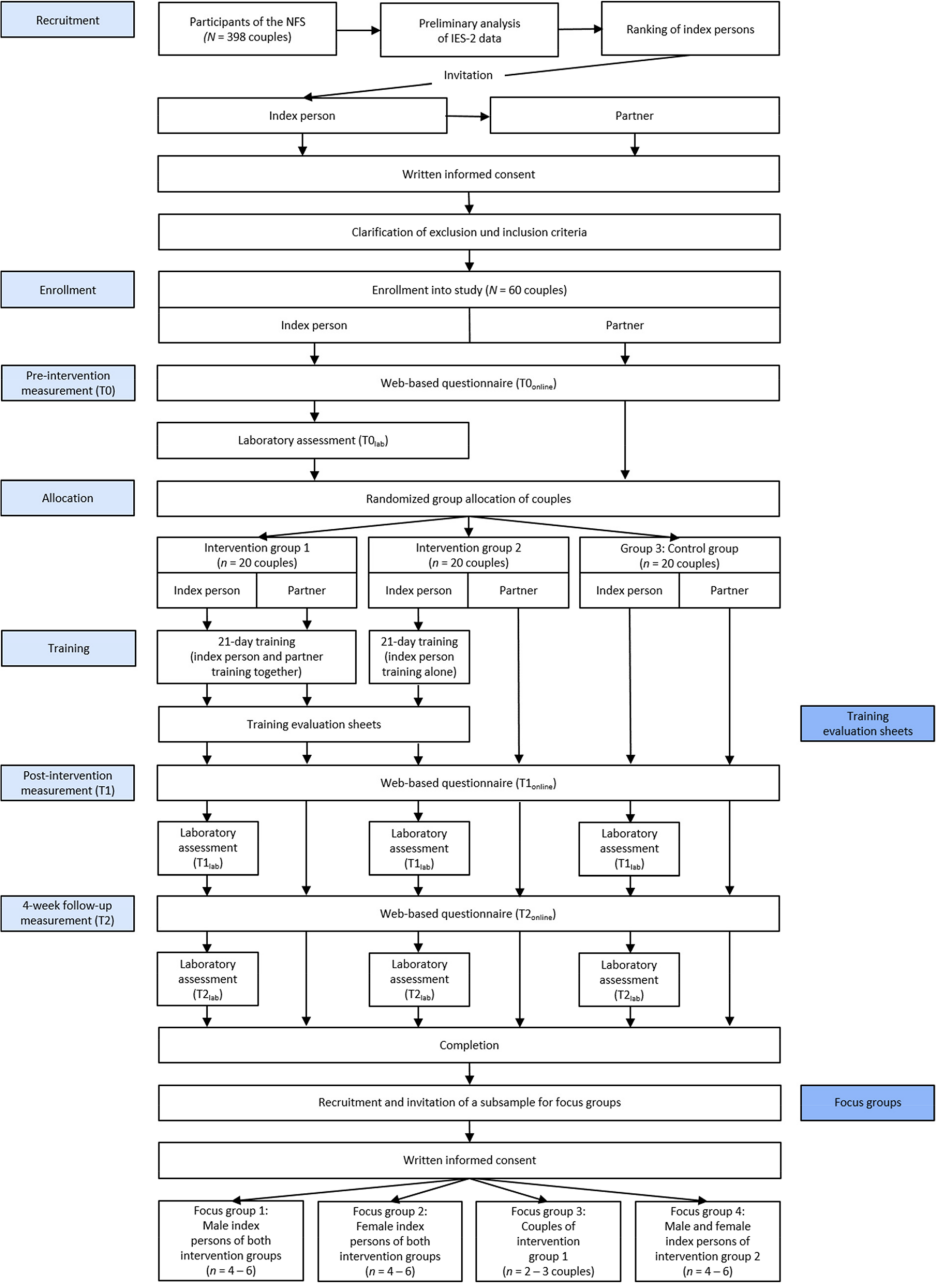
Flow chart of the study design

This pilot randomized study is nested into the Nutritional Intervention for Healthy Aging (NutriAct) Family Study (NFS). The NFS is part of a competence cluster funded by the German Federal Ministry of Education and Research [42]. The intervention is based on a within-subject design with three measurement points (pre-intervention [T0], post-intervention [T1], 4-week follow-up [T2]), and a 21-day training period. A three-arm intervention study design is used to compare two different intervention groups (training together as a couple vs. training alone) with a control group. Each measurement point includes a web-based survey that measures different self-reported indicators and is completed by both partners. In addition, the preassigned index person of each couple is tested at all three measurement points in the laboratory (i.e., examination center) of the Human Study Center (HSZ) of the German Institute of Human Nutrition Potsdam-Rehbruecke. Moreover, a training evaluation (via training evaluation sheets) as well as four qualitative focus group discussions – each with four to six participants – will be conducted to evaluate participants’ acceptance as well as the medium-term impact of the training and its implementation as a new behavioral routine in everyday life. A detailed overview of the study procedure is illustrated in Fig. 2.

The present study protocol was written in accordance with the Standard Protocol Items: Recommendations for Interventional Trials (SPIRIT) reporting guidelines [43]. The SPIRIT checklist is provided as Additional file 1.

### Participants and sample size

A total of *N* = 60 couples aged 50–80 years is to be recruited as a first test of the training feasibility and effectiveness. Eligible for inclusion are heterosexual couples who are living together and willing to participate in a randomized controlled trial. Since the intervention focuses on the promotion of intuitive eating behavior, participants with comparatively lower levels of intuitive eating have a high priority for invitation to participate in the randomized controlled trial (see next paragraph). Exclusion criteria include kidney diseases, gastrointestinal diseases, cognitive impairments, and cardiovascular problems (especially wearing a cardiac pacemaker).

### Recruitment

Study participants are recruited from the NFS, a web-based prospective study on the basis of food choice from epidemiological, psychological, and sociological perspectives [42]. The NFS is an ongoing interdisciplinary study that examines a variety of potential factors influencing food choice based on the DONE framework [44].

As the newly developed, digital, mindfulness-focused training is intended to support those people with poor intuitive eating behavior to better perceive their physical signals and react to them appropriately, a selective prevention approach is being pursued. Therefore, based on already available data of the NFS, people were ranked according to their levels of intuitive eating behavior. In a sub-sample of *n* = 398 eligible couples [45], the partner who, according to the Intuitive Eating Scale-2 (IES-2) [4], reported the lower level of intuitive eating was the preferred index person. These were then ranked according to the level of intuitive eating and invited to participate in the RCT. The index persons with their respective partners are successively recruited based on their IES-2 rank, beginning with those index persons who reported the lowest level of intuitive eating.

Index persons are initially contacted by mail and invited to participate in the study together with their partners. Prior to inclusion into the study, both the index person and their partner provide written informed consent. Index persons are then contacted by phone to clarify inclusion and exclusion criteria. Successive recruitment for this ongoing study, i.e., the arrangement of appointments for the assessments in the examination center, started in October 2020.

### Study procedure

**Fig. 3:**
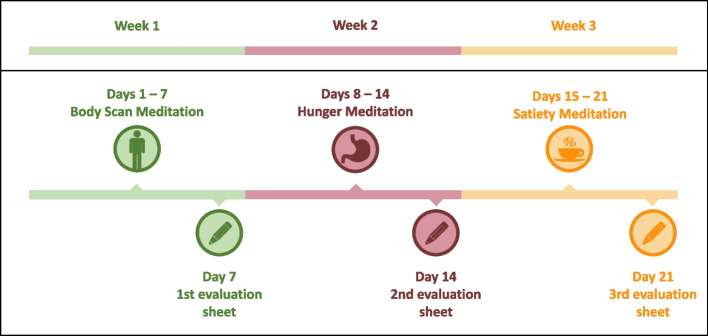
Training schedule

Once included in the study, index persons receive a personalized time schedule for their three laboratory appointments. Moreover, all participants receive an email that contains a link to the pre-intervention online survey (T0_online_). Both the index person and their partner are asked to complete the online questionnaire before the index person’s first laboratory appointment. As a reminder and to clarify any questions, index persons are contacted by phone the day before the assessment in the laboratory (T0_lab_). After completing both pre-intervention measures (T0_online_ and T0_lab_), index persons receive a personalized sealed folder that initially informs them about the group they and their partner were randomly assigned to. Folders for intervention group participants (groups 1 and 2) contain information about the training, detailed training instructions, as well as training evaluation sheets for each exercise that participants are asked to complete after the training (see Fig. 3). Intervention group participants also receive a USB flash drive that contains the intervention material (audio files for each exercise) and a 20-minute introductory video about the study, the training, and its theoretical background. To keep study assistants blinded when handing out the folders and for attention control, control group participants (group 3) also receive a personalized sealed folder. It contains different crossword puzzles and a USB flash drive with an audiobook file (“The Chimes” by Charles Dickens, [46]) that participants are free to listen to.

Three weeks after completing the pre-intervention measures, all participants receive another personalized email that contains a link to the post-intervention online survey (T1_online_), asking for completion before the second laboratory assessment (T1_lab_) of the index person. Again, index persons are contacted by phone the day before T1_lab_ to clarify any questions. Following the same procedure, follow-up measures (T2_online_ and T2_lab_) take place 4 weeks after completion of the post-intervention measures. As compensation for travel expenses, index persons receive €10 for each assessment in the laboratory.

### Intervention groups and control group

Couples are randomly assigned to one of the two intervention arms (groups and 2) or a control group (group 3). Both intervention groups only differ with respect to how they implement the training in everyday life: Index persons of group 1 are instructed to complete the training with their partners. No further instructions are given regarding the exact implementation of training together. Index persons of group 2 are instructed to complete the training by themselves, i.e., without their partner. Participants of the control group (group 3) do not complete any training. However, after finalizing the follow-up measures, the control group is given the opportunity to receive the training as well.

### Training in both intervention groups

The training consists of three different mindfulness-based audio exercises (body scan mediation, hunger meditation, satiety meditation), which participants are asked to perform daily over a period of 21 days. All exercises are provided as guided meditation audio files. In line with common recommendations [30], participants are asked to complete the exercises in a quiet place and avoid possible distractors (e.g., mobile phone, radio, television). Exercises should serve as a timeout from the daily routine, eyes should be closed during the exercises, upcoming thoughts or experiences should not be judged or criticized. Participants decide on their own at what time of day they want to practice. Participants are instructed to perform each exercise on seven consecutive days. All audiotapes include a short introduction followed by the specific exercise sequence and a short wake-up phase.

The first exercise (week 1) is a body scan meditation edited from a script by Kabat-Zinn and Valentin [47], based on the procedure described by Fischer and colleagues [30]. During this 20-minute exercise, participants are asked to lie down and focus their attention on different parts of the body, starting with the feet and moving up slowly to the top of the head. There is evidence that interoception can be improved by interventions that are based on body scan meditation [30]. Both the second and the third exercise are based on the intuitive eating woorkbook by Tribole and Resch [48]. During the second week, participants are instructed to perform a hunger meditation *before* a meal. During this 8-minute exercise, participants are first asked to sit comfortably. Subsequently, they are instructed to mindfully focus on the perception of hunger signals, such as their location and intensity as well as the type of sensation. The third exercise (week 3) is a satiety mediation that participants are asked to perform *after* a meal and, again, in a comfortable sitting position. During this 9-minute exercise, participants are instructed to focus on the perception of satiety signals, evaluate the extent of their satiety, and notice whether and how they feel satiety in their stomach or other body parts. A training schedule is presented in Fig. 3.

Following the review of Schuman-Olivier et al. [49], the main skills/strategies that are targeted by the training are self-related processes (interoceptive awareness, self-efficacy, self-critical rumination and self-monitoring). Furthermore, the training also includes aspects of emotion regulation (emotion differentiation, decentering) and attentional / cognitive control (volitional orienting, alerting, conflict monitoring and inhibitory control).

To evaluate participants’ adherence to instructions, the post-intervention online survey (T1_online_) contains an additional group-specific item that serves as a manipulation check (e.g., group 2: “Did you actually do the training alone during the last 21 days or did you ask for or receive support from your partner?”; 1 = *alone*; 2 = *with my partner*).

### Group allocation, blinding, and confidentiality

Groups are allocated using ID numbers (between 1 and 75) that were randomly assigned to the three groups prior to recruitment via the online software Research Randomizer (version 4.0) [50]. ID numbers (and thus the respective group) are assigned to the participants in the order in which they are included in the study. The random assignment of numbers ensures that no conclusions can be drawn between ID numbers and groups.

All participants are blinded throughout the pre-intervention measurements, as these precede the distribution of the sealed folders that contain information about the participants’ group (see paragraph ‘study procedure’). Participants are instructed to maintain strict silence about their group allocation during the post-intervention and follow-up measurements. Study assistants are blinded during all laboratory assessments. To ensure unbiased ascertainment and analysis of outcomes, data collectors, data managers (research personnel handling data coding and cleaning) as well as data analysts are blinded.

To maintain participant confidentiality, all study-related information will be stored securely at the HSZ of the German Institute of Human Nutrition Potsdam-Rehbruecke. All records that contain names or other personal identifiers, such as informed consent forms, will be stored separately from study records identified by ID numbers. All databases will be secured with password-protected access systems.

### Study measures

**Fig. 4:**
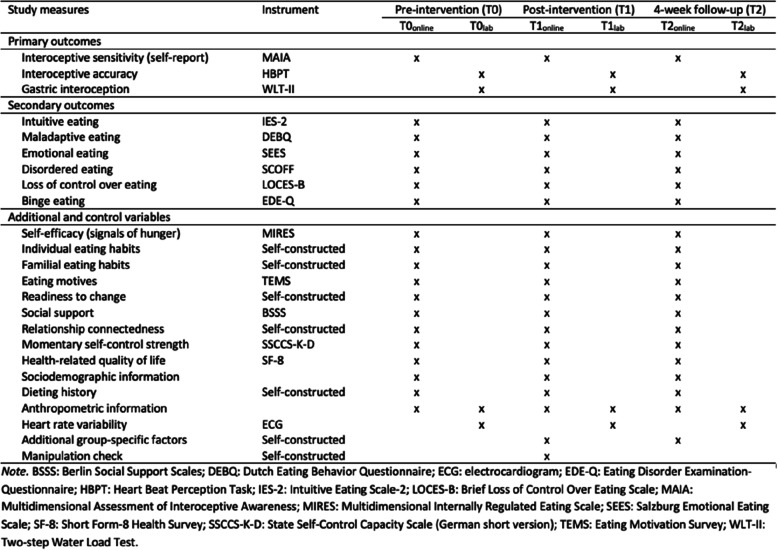
Overview of study measures (SPIRIT figure). *Note.* BSSS: Berlin Social Support Scales [52]; DEBQ: Dutch Eating Behavior Questionnaire [53]; ECG: electrocardiogram; EDE-Q: Eating Disorder Examination-Questionnaire [54]; HBPT: Heart Beat Perception Task [55]; IES-2: Intuitive Eating Scale-2 [4]; LOCES-B: Brief Loss of Control Over Eating Scale [56]; MAIA: Multidimensional Assessment of Interoceptive Awareness [57]; MIRES: Multidimensional Internally Regulated Eating Scale [58]; SCOFF: SCOFF [59]; SEES: Salzburg Emotional Eating Scale [60]; SF-8: Short Form-8 Health Survey [61]; SSCCS-K-D: State Self-Control Capacity Scale (German short version) [62]; TEMS: Eating Motivation Survey [63]; WLT-II: Two-step Water Load Test [64]

This study includes a web-based assessment of various psychological constructs at three measurement points (T0_online_, T1_online_, T2_online_). The comprehensive online test battery is to be completed by both the index person and their partner of all three groups. The respective online questionnaires were generated using the online software SoSci Survey [51]. In addition, several objective measurements of the index persons are assessed at all three measurement points (T0_lab_, T1_lab_, T2_lab_) in the laboratory. Partners were not invited to attend the laboratory assessments. For a detailed overview of all study measures (SPIRIT figure), see Fig. 4.

#### Primary outcome

It has been suggested that the ability to perceive and process internal bodily signals comprises different dimensions of both subjective and objective interoceptive sensitivity [65]. Therefore, both a self-report measure and two objective measures are used to assess interoceptive sensitivity as the study’s primary outcome. To objectively quantify individual differences in behavioral interoceptive performance, also referred to as interoceptive accuracy (IA) [65], objective measures of both cardiac and gastric interoceptive ability will be assessed.

#### Interoceptive sensitivity

As part of the online questionnaires, participants’ subjective beliefs (self-report) in their interoceptive ability will be assessed with the German version [66] of the Multidimensional Assessment of Interoceptive Awareness (MAIA [57];). The 32-item multidimensional instrument assesses different aspects of interoceptive body awareness with eight subscales (*Noticing, Non-Distracting, Not-Worrying, Attention regulation, Emotional awareness, Self-Regulation, Body listening, Trust*). Each item (e.g., “I am able to consciously focus on my body as a whole.”) is rated on a 6-point Likert scale ranging from *never* (= 0) to *always* (= 5). Previous research has supported the scale’s validity and internal consistency, with Cronbach’s α ranging between .56 and .89 for the different subscales [66].

#### Cardiac interoceptive accuracy

Cardiac interoceptive accuracy is measured using the well-established Heart Beat Perception Task (HBPT [55];). During this task, participants are positioned on an adjustable examination couch and asked to sit in a comfortable, semi-upright position. They are instructed to listen to and silently count their own heartbeats (intervals of 25, 35, 45, and 100 seconds, respectively) without using any additional aid (e.g., taking their pulse manually). Participants are not aware of the lengths of the intervals, and their order of presentation is counterbalanced across participants. Simultaneously, an electrocardiogram (ECG) of the objective heartbeats is recorded using a heart rate variability (HRV) scanner from BioSign GmbH, with clamp electrodes placed at both wrists (sampling rate of 500 Hz, 16-bit resolution). An IA score is calculated using the following commonly used formula:

$$\textrm{IA}\ \textrm{score}=\frac{1}{4}\sum \Big(1-\frac{\left(\left|\textrm{recorded}\ \textrm{heartbeats}-\textrm{counted}\ \textrm{heartbeats}\right|\right)}{\textrm{recorded}\ \textrm{heartbeats}}$$

IA scores range between 0 and 1, with higher scores indicating a smaller difference between recorded and reported heartbeats, i.e., a higher interoceptive accuracy.

#### Gastric interoceptive accuracy

As a domain-specific measure of interoceptive accuracy, gastric interoception is measured using the two-step Water Load Test (WLT-II [64];). Following the procedure by van Dyck and colleagues [64], participants are instructed to drink still water at room temperature over two successive 5-minute intervals. During the first phase, participants are asked to drink water until reaching the point of perceived satiation. The first phase is followed by a second one, where participants are instructed to drink water until reaching the point of maximum stomach fullness. Participants are not informed that there will be a second drinking phase to rule out any influence on the amount of water consumed during the first phase. Participants are instructed to drink the water through long straws from non-transparent 5-l flasks that are filled with 1.5 l of water. Participants receive a refilled flask for each drinking phase. After giving the instructions, study assistants leave the room during each phase. After each phase, the amount of water consumed (in milliliters) is recorded in another room. Participants are instructed to refrain from eating and drinking at least 2 hours before the session and are encouraged to use the toilet before being tested.

Different WLT-II scores can be calculated. In addition to the amount of water consumed up to satiation (step 1: water volume required to produce satiation [sat_ml]) and maximum fullness (step 2: additional water volume required to produce maximum fullness [Δfull_ml]) and the total water volume (total_ml), an individual index of gastric interoception (sat_%) will be calculated. It is defined as the percentage of satiation to maximum fullness and it is not confounded with stomach capacity. It is calculated using the following formula [64]: $\textrm{sat}\_\%=\frac{\textrm{sat}\_\textrm{ml}}{\textrm{total}\_\textrm{ml}}\ \textrm{x}\ 100.$

In addition, subjective sensations related to the WLT-II are assessed using the WLT-II questionnaire [64]. Before and after each drinking phase, participants are asked to complete 8 items, rating their momentary feelings of satiation and fullness as well as sensations of discomfort, guilt, sluggishness, nausea, and arousal. Items are rated on a 7-point Likert scale that ranges from *no sensation/not at all* (= 1) to *extremely* (= 7). According to van Dyck and colleagues [64], the WLT-II shows satisfactory repeatability over time for both drinking periods and proves to be a reliable and valid instrument for assessing gastric interoception.

### Secondary outcomes

#### Intuitive eating

Intuitive eating is measured with the German version [9] of the Intuitive Eating Scale-2 (IES-2 [4];). The scale assesses the four aspects of intuitive eating: *Unconditional permission to eat* (UPE, 6 items, e.g., “If I am craving a certain food, I allow myself to have it.”), *Eating for physical rather than emotional reasons* (EPR, 8 items, e.g., “I find other ways to cope with stress and anxiety than by eating.”), *Reliance on hunger and satiety cues* (RHSC, 6 items, e.g., “I rely on my hunger signals to tell me when to eat.”), and *Body-food choice congruence* (B-FCC, 3 items, e.g., “I mostly eat foods that give my body energy and stamina.”). All 23 items are rated on a 5-point Likert scale ranging from *strongly disagree* (= 1) to *strongly agree* (= 5). Therefore, higher mean values reflect higher levels of intuitive eating. Previous research has supported the scale’s validity as well as its internal consistency for the total score (Cronbach’s α = .89) and each of the four subscales, with Cronbach’s α ranging from .73 to .91 [9].

#### Maladaptive eating

Ratings of maladaptive eating are obtained from the German version [67] of the Dutch Eating Behavior Questionnaire (DEBQ [53];), a widely used measure of eating styles. Responses to its 30 items are rated on a 5-point Likert scale that ranges from *never* (= 1) to *very often* (= 5). Three subscales reflect the different eating styles of restraint eating (10 items, e.g., “I deliberately eat less in order not to become heavier.”), emotional eating (10 items, e.g., “I have the desire to eat when I am feeling lonely.”), and external eating (10 items, e.g., “I eat more than usually when I see others eating.”). Previous research [53, 67] has supported the scale’s validity and the subscales’ reliability, with Cronbach’s α of the three subscales ranging between α = .87 and .95.

To be able to not only measure emotional eating as a coping strategy to deal with negative emotions as in the DEBQ, but also to assess emotional eating related to negative as well as positive emotions, the Salzburg Emotional Eating Scale (SEES [60];) was used. The SEES allows for a differentiation between four types of emotions (Happiness, sadness, anger, anxiety) and between increased or decreased food intake in response to emotions. It consists of 20 items that each begin with the stem “When I feel/am …” , followed by an adjective describing an emotion. Response categories range from 1 (*I eat much less than usual*) to 5 (*I eat much more than usual*). Internal consistency of the four subscales ranges between Cronbach’s α = .71 and .87 in different samples [60].

#### Maladaptive and pathological eating

Disordered eating behavior is assessed using the German version [68] of the SCOFF [59]. The 5 items (e.g., “Do you believe yourself to be fat when others say you are too thin?”) are rated on a dichotomized scale (*yes* or *no*), with a total score of ≥ 2 as a cutoff point to select persons at risk of developing an eating disorder. In a validation study, Cronbach’s α was .66 [68].

Loss of control over eating is measured using a German translation of the Brief Loss of Control Over Eating Scale (LOCES-B [69];). The original 7 items (e.g., “I continued to eat past the point when I wanted to stop.”) were translated into German by two independent psychologists and then back-translated, following the WHO guidelines [70]. On a 5-point Likert scale ranging from *never* (=1) to *always* (=5), participants rate how often they experienced loss of control during the past 28 days. The scale’s internal consistency reached Cronbach’s α = .93 in the original validation study [69].

Binge eating is measured with the following two items of the German version [54] of the Eating Disorder Examination-Questionnaire (EDE-Q [71];): (a) “Over the past 28 days, how many times have you eaten what other people would regard as an unusually large amount of food (given the circumstances)?” and (b) “On how many of these times did you have a sense of having lost control over your eating (at the time you were eating)?”. Participants are asked to rate the frequency of binge eating in terms of number of episodes in the past 28 days, with higher scores reflecting higher levels of binge eating.

### Additional and control variables

#### Self-efficacy in using physiological signals of hunger

Self-efficacy in using physiological signals of hunger is assessed using a German translation of the eponymous subscale of the Multidimensional Internally Regulated Eating Scale (MIRES [58];). Following the WHO guidelines [70], the original items were translated into German by two independent psychologists and then back-translated. Its 6 items (e.g., “I find it easy to let my hunger determine when I eat.”) are rated on a 7-point Likert scale, ranging from 1 (*completely untrue for me*) to 7 (*completely true for me*). In the original validation study, the composite reliability was .90 [58].

#### Individual and familial eating habits

Individual eating habits and routines during mealtime are assessed using 9 items [72]. The items (e.g., “I chew my food thoroughly.”) are rated on a 5-point Likert scale that ranges from *never* (= 1) to *always* (= 5). Familial eating habits and eating traditions [42] are measured with 15 items (e.g., “Food is very important to our family.”) that are rated on a 5-point Likert scale ranging from *strongly disagree* (= 1) to *strongly agree* (= 5). In a preliminary study [9], internal consistency of this scale reached Cronbach’s α = .76.

#### Eating motives

Motives underlying eating behavior are measured using the following three subscales of the Eating Motivation Survey (TEMS [63];): (a) *Liking* (5 items; e.g., “I eat what I eat, because I think it’s delicious.”), (b) *Need and Hunger* (4 items, e.g., “I eat what I eat, because I’m hungry.”), and (c) *Pleasure* (5 items, e.g., “I eat what I eat, because I enjoy it.”). Items are rated on a 7-point Likert scale that ranges from *never* (= 1) to *always* (= 7). Previous research [63] has supported the scale’s validity and the subscales’ reliability, with Cronbach’s α = .84 (*Liking*), and .77 (*Pleasure*). The internal consistency of the *Need and Hunger* subscale was comparably low, with Cronbach’s α = .48 (*Need and Hunger*).

#### Readiness to change

Self-generated items are used to evaluate participants’ readiness to change towards a more mindful and intuitive approach to eating. Items were generated based on the *Transtheoretical Model* (TTM [73];) that conceptualizes behavior change as a process that involves progression through different stages of change (SOC). Readiness to change is assessed by four items (e.g., “When I eat, I consciously pay attention to my bodily signals (e.g., feelings of hunger or satiety).”). Following Prochaska and DiClemente [74], a six-choice response format is applied to categorize participants into the six different stages of change (e.g., 1 = *No, and I do not intend to start doing so within the next 6 months* for the precontemplation stage).

#### Social relationships

Social support is measured with the 11-item *Actually received social support* subscale of the Berlin Social Support Scales (BSSS [52];), a frequently used measure of different cognitive and behavioral aspects of social support. Respondents rate on a 4-point Likert scale how much emotional, instrumental, and informational social support they have received by their partners during the past weeks (e.g., “My partner encouraged me not to give up.”; *strongly disagree* (= 1) to *strongly agree* (= 4)). The scale’s internal consistency was Cronbach’s α = .83 in the original validation study [52].

Relationship connectedness describes the degree of belonging and relatedness between a person and their partner [75] and is measured using the item “How closely connected do you feel today with your partner?” [45]. Response categories range from 1 (*not close at all*) to 5 (*very close*) on a 5-point Likert scale.

#### Momentary self-control strength

Momentarily available self-control strength is measured by a German short version (SSCCS-K-D [62];) of the State Self-Control Capacity Scale (SSCCS [76];). Its 10 Items (e.g., “I feel sharp and focused.”) are rated on a 7-point Likert scale ranging from *not true at all* (= 1) to *very true* (= 7). In the validation study, Cronbach’s α was .87 [62].

#### Health-related quality of life

Health-related quality of life is measured using the German version [77] of the Short Form-8 Health Survey (SF-8 [78];), which is an abbreviated version of the original SF-36 Health Survey (SF-36 [61];), a generic instrument to measure perceived health status. The SF-8 measures eight domains (response categories) with one item each, including general health (1 = *excellent*, 6 = *very poor*), physical functioning (1 = *not at all*, 5 = *could not do physical activities*), role physical (1 = *none at all*, 5 = *could not do daily work*), bodily pain (1 = *none*, 6 = *very severe*), vitality (1 = *very much*, 5 = *none*), social functioning (1 = *not at all*, 5 = *not do social activities*), mental health (1 = *not at all*, 5 = *extremely*), and role emotional (1 = *not at all*, 5 = *could not do daily activities*). Using a norm-based scoring method [78], Physical Component Summary (PCS) and Mental Component Summary (MCS) measures can be calculated by weighting each item, with higher summary PCS and MCS scores indicating better health.

#### Sociodemographic information and dieting history

Information on sex and age are collected as self-reports at all three measurement points. Dieting history is assessed using the following three items [9]: (a) “Are you currently dieting (e.g., eating less than usual, eating only specific foods, skipping meals, fasting …)? ” – *yes* or *no*; (b) “Have you ever dieted?” – *yes* or *no*; (c) if the answer to the previous item is yes: “How often?” – *number of diets*. Based on these items, participants can be classified into three dieting groups (never, former dieters, current dieters).

#### Anthropometric information

Information on body height and body weight is collected online as self-reports at all three measurement points. In addition, study assistants measure the participants’ height and weight with calibrated instruments (a stadiometer by Seca and a MS 4202 medical floor scale by Marsden) in the laboratory. Participants are weighed and measured in light clothing and without shoes.

#### Heart rate variability

Heart rate variability (i.e., the variation in time between consecutive heartbeats) is considered a biological marker of self-regulation [79]. To assess heart rate variability at rest, an electrocardiogram (ECG) is recorded using the HRV scanner from BioSign GmbH, with clamp electrodes placed at both wrists (sampling rate of 500 Hz, 16-bit resolution). Participants are instructed to sit quietly in a comfortable, semi-upright position for 5 minutes and breathe normally. Pre-processing of data and score calculation will be performed with Kubios HRV software (version 3.5) [80]. The standard deviation of normal-to-normal RR intervals (SDNN) as a commonly used time-domain HRV index [65] will be calculated to quantify the amount of HRV between successive heartbeats.

#### Additional group-specific measures

T1_online_ and T2_online_ surveys comprise additional group-specific items that are used to measure intervention-related factors, such as subjective training success (“Overall, do you feel that your ability to perceive hunger and satiety signals has improved because of the training?”; 1 = *not at all*, 6 = *very much*) and recommendation of training (“Would you recommend the training to others?”; 1 = *not at all*, 6 = *absolutely)*.

### Intervention evaluation and acceptance

To assess participants’ overall acceptance of the training, its feasibility as well as its medium-term impact and (non-) continuation in everyday life, a two-step evaluative strategy consisting of training evaluation sheets as well as focus group discussions will be executed.

#### Training evaluation sheets

Intervention group participants are instructed to complete three self-constructed paper-pencil training evaluation sheets. Participants receive three largely identical questionnaires (one for each exercise) that are to be completed after each week of the training (see Fig. 3). Questionnaires comprise 19 items as well as 3 additional group-specific items for group 1 participants. Items assess different subjective evaluative aspects of the three exercises, such as comprehensibility, feasibility, user-friendliness, implementation into everyday life, and perceived barriers. All items are rated either on 5-point Likert scales or in an open-ended response format.

#### Focus group discussions

To explore participants’ acceptance of each exercise as well as their overall experiences across time, four qualitative focus group discussions [81] will be conducted at least 3 months after the follow-up measures. For the four qualitative focus group discussions (see Fig. 2), approximately *n* = 16–24 participants are drawn from the total sample (*N* = 60 couples). To allow for comparative qualitative analyses, two groups will ideally involve couples, and the others are planned with individuals who have completed the training either alone or together with their partner. The latter two groups are to be conducted separated by gender to allow for potential gender-related effects.

A semi-structured guideline will be developed to stimulate discussion and support the exchange of experiences among participants. This guideline will be informed by first descriptive results of the above-mentioned training evaluation sheets. The focus of the guideline lies on the overall intervention as well as specific experiences with the three training exercises (body scan mediation, hunger meditation, satiety meditation) over the course of time and within the couple context. For example, questions will address the influence and support of the partner in the implementation of the exercises. In addition, the guiding questions are aimed at the general potential for change in connection with intuitive eating, e.g., the extent to which everyday practices have changed by the time of the focus groups. The guideline will be adjusted according to the group composition (couples vs. individuals).

The focus groups will be recorded and the audiotapes will be transcribed. Each group discussion takes place at the German Institute of Human Nutrition Potsdam-Rehbruecke. Index persons are contacted via phone to clarify their readiness to participate. In case of agreement, participants receive detailed study information via email. Prior to the group discussions, each participant provides written informed consent – including consent for being audiotaped. Participation is compensated with an expense allowance of €20 per person.

### Data management and monitoring

Data of all online questionnaires (T0_online_, T1_online_, T2_online_) will be collected electronically using the secure online software SoSci Survey [51]. Data of all laboratory assessments (T0_lab_, T1_lab_, T2_lab_) will be entered electronically by the trained study assistants, using the Study Management System (SMS) of the HSZ. Data of the paper-pencil training evaluation sheets will be transferred to an electronic database by experienced data entry personnel via the online software SoSci Survey. All research data will be stored on a secure, password-protected computer server that will only be accessible to the research team. Range checks for all data values will be carried out to promote data quality. Web-based data collection and adherence to a detailed study manual within the laboratory assessments ensure standardization of data collection and thus further promote the quality of all research data.

Due to the health-promoting focus and preventive approach of the present study, no negative side effects are expected. Therefore, no stopping rules are defined. Moreover, no interim analyses are intended.

### Analyses

#### Hypothesis testing

Data will be analyzed using the per-protocol (PP) analysis. The study encompasses three measurement points, of which two are post-intervention. First, descriptive analyses will be performed to describe the baseline demographic characteristics, stratified by intervention group. Correlational patterns will be analyzed and requirements for statistical analyses will be checked. Our main analyses will be performed using Bayesian methods of inference. Following Wagenmakers and colleagues [82], Bayesian inference entails several advantages (such as its application to all sample sizes) compared to classical inference and thus represents a beneficial alternative to classical approaches. Using the Bayes factor, evidence for the alternative hypothesis (H_1_) is compared with the evidence for the null hypothesis (H_0_). Thereby, evidence for both hypotheses (H_1_ and H_0_) can be quantified from the observed data, which is another advantage of Bayesian inference compared to classical approaches [82]. If necessary, our analyses will be corroborated by classical null hypothesis significance testing using *p* values.

In order to investigate whether the training improved interoceptive sensitivity and intuitive eating and decreased maladaptive eating behaviors of the index persons from pre-intervention to follow-up, repeated measurements analyses will be performed for each construct of interest using multivariate linear regression models to determine intergroup effects, adjusting for the baseline values of the respective outcome variables. To analyze the training effect on interoceptive sensitivity (primary outcome), intervention group participants (group 1 and group 2) will be compared to control group participants (group 3) in terms of their MAIA scores as well as their interoceptive accuracy scores (HBPT) and WTL-II scores. Intervention group participants and their spouses will be compared to control group participants and their spouses in terms of their IES-2 and DEBQ subscale scores in order to analyze the training effect on different eating behaviors (secondary outcome). To test whether targeting couples enhances the effectiveness of the training, participants completing the training together with their spouses (group 1) will be compared to participants performing the training alone (group 2) and control group participants (group 3). Furthermore, correlative patterns between additional variables and outcome variables will be analyzed to be able to add relevant variables to the models. Also, exploratory analyses on the role of spouses within this intervention will be carried out.

Missing data per measurement point and over time are handled by full information maximum likelihood estimation (FIML [83];), a model-based statistical approach for handling missing data that produces unbiased parameter estimates and standard errors if data are missing at random. Studies have shown that FIML outperforms traditional approaches for handling missing data and performs as well as multiple imputation [84]. The analyses will be conducted using IBM SPSS Statistics 27, Mplus 7 [85], and JASP [86].

#### Intervention evaluation and acceptance

In order to gain in-depth insights into the short- and midterm acceptance of the training, its feasibility and its implementation into daily life, the quantitative assessments of the training evaluation sheets and qualitative focus group discussions will be triangulated in a Sequential Convergence Mixed-Methods Design [87]. First, the training evaluation sheets will be descriptively analyzed. The results will inform the development of the qualitative guideline. Subsequently, each data base will be analyzed separately. The focus group discussions will (comparatively) be analyzed by means of Qualitative Content Analysis [88]. Finally, to triangulate the findings of each part, the quantitative results will be transformed into qualitative themes [87].

## Discussion

Eating in absence of hunger is quite common and associated with increased food intake and poorer nutritional food choices [89]. In addition, there is evidence that deficits in interoception are related to a higher body weight status [21]. Therefore, training the ability to accurately perceive one’s own internal hunger and satiety signals might be an effective method to increase eating in accordance with physiological signals and thus to promote an adaptive style of eating [28] and a body weight status of low health risks. Despite the high prevalence of body weight- and eating-related problems among older-aged adults [32], this group is often neglected in research and practice [34]. The present pilot randomized intervention study aims at investigating the feasibility and effectiveness of a 21-day mindfulness-based training program to increase interoceptive sensitivity in a sample of older couples.

The findings of this study will further extend our current knowledge in many ways. First, this study takes an innovative and holistic approach as it regards health-promoting eating as part of a health-oriented and mindful lifestyle in older age. The main focus of our research is to develop and test a program that explicitly focuses on promoting adaptive eating behavior. Accordingly, the effectiveness of the training program will be evaluated based on its hypothesized effect on more adaptive eating behavior and improved interoceptive awareness. The latter is considered the essential mechanism of action to build intuitive eating behavior and, in turn, to maintain or reach a health-promoting nutrition and body weight with lower health risks. We developed an easy-accessible digital intervention by providing guided audiotapes that can be easily implemented in everyday life and therefore follow a (selective) prevention approach. Second, this study takes an interdisciplinary approach by combining both quantitative and qualitative methods and broad disciplinary expertise. The mixed-methods evaluation approach will allow us to get a deeper insight into the barriers and facilitators in implementing a low-threshold digital intervention in this age group. These data will provide the empirical basis to modify the training approach in order to better meet the needs of the participants. Third, we will use a comprehensive set of validated instruments that assesses a broad range of potentially relevant constructs. Both a self-report measure and objective measures will be used to assess interoceptive sensitivity as the study’s primary outcome. Fourth, focusing on middle-aged and older adults, we will investigate a population that was mainly neglected in research on eating behavior so far [34]. In fact, most previous research focuses on individual influences on dietary intake and eating behavior; based on a socio-ecological perspective, more attention should be paid to the social influences on eating behavior, particularly from the viewpoint of interpersonal relationships. Close social relationships may play a larger role than regarded so far. Therefore, this study will give first insights into the usefulness and feasibility of partner-based interventions in older adults. In an exploratory way, our results will also allow deeper insights into the potential benefits of partner-focused interventions compared to individual interventions in order to promote healthier eating styles.

There are some possible limitations of the present study, including a relatively small sample size. However, Bayesian methods of inference are equally valid for all sample sizes [82]. Moreover, the sample size will be large enough to get first insights into the training effectiveness and to investigate its feasibility. Another limitation concerns the representativeness of the sample. Participants are to be recruited from the participants of the NFS [42], drawn from the European Prospective Investigation into Cancer and Nutrition (EPIC)-Potsdam Study [90]. Thus, participants will be relatively experienced in terms of participating in research studies and potentially biased towards a generally higher interest in nutrition-related topics. In addition, the NFS participant pool shows a comparably high level of education [45], which might further limit the representativeness of the sample. As the enrollment in the present study depends on the agreement and cooperation of both partners, there might also occur a selection bias towards functional relationships. In addition, following a selective prevention approach, the eligibility of participants will be pre-selected based on their level of intuitive eating. This allows to better analyze the general suitability of the intervention in relation to the improvement of the outcomes of interest, but at the same time limits the generalizability of the results (e. g., with regard to universal prevention strategies). Furthermore, partners were not invited to the laboratory assessments as the focus of the study was to investigate the feasibility of our training and how the training influenced the index person. However, transfer effects might be interesting to investigate. Lastly, as this is a pilot RCT, further research is needed to enrich the evaluation of our intervention and to investigate the effects of this intervention more in-depth (e.g., with a longer follow-up period, by assessing relevant mechanisms of how partners influence each other regarding health-promoting eating behavior and by also assessing actual food intake).

In conclusion, our study will provide first insights into both the feasibility and effectiveness of a mindfulness-based training program and allow us to analyze the postulated mechanism of action (interoception). Thereby, it will contribute to a deeper understanding of how to promote healthy eating in older age at a community level, thereby benefitting healthy aging.

### Dissemination plans

Findings from this research will be widely disseminated through conference papers, research reports, and academic publications in peer-reviewed journals. In addition, study participants will be informed about the study results via a newsletter. Authorship for future publications presenting the results of the present study will be determined in accordance with ICMJE guidelines. There is no intended use of professional writers.

## Supplementary Information

**Additional file 1.**

## Data Availability

All Principal Investigators will be given access to the cleaned data sets. As this study is part of the ongoing NFS, public access to data will be arranged on a reasonable request and with the permission of all collaboration partners.

## Abbreviations

BSSS: Berlin Social Support Scales
DEBQ: Dutch Eating Behavior Questionnaire
ECG: Electrocardiogram
EDE-Q: Eating Disorder Examination-Questionnaire
EPIC: European Prospective Investigation into Cancer and Nutrition Potsdam Study
FIML: Full information maximum likelihood estimation
HBPT: Heart Beat Perception Task
HRV: Heart rate variability
IA: Interoceptive accuracy
IES-2: Intuitive Eating Scale-2
LOCES-B: Brief Loss of Control Over Eating Scale
MAIA: Multidimensional Assessment of Interoceptive Awareness
MBI: Mindfulness-based intervention
MIRES: Multidimensional Internally Regulated Eating Scale
NFS: NutriAct Family Study
SEES: Salzburg Emotional Eating Scale
SF-8: Short Form-8 Health Survey
SSCCS-K-D: State Self-Control Capacity Scale (German short version)
TEMS: Eating Motivation Survey
T0: pre-intervention
T1: post-intervention
T2: 4-week follow-up
WLT-II: Two-step Water Load Test
